# Interactions of Amphiphilic Janus Nanoparticles with Lipid Monolayers

**DOI:** 10.1021/acs.langmuir.5c06024

**Published:** 2026-02-12

**Authors:** Kolattukudy P. Santo, Younjin Min, Alexander V. Neimark

**Affiliations:** 1 Department of Chemical and Biochemical Engineering, Rutgers, The State University of New Jersey, Piscataway, New Jersey 08854, United States; 2 Department of Chemical and Environmental Engineering, 8790 University of California Riverside, Riverside, California 92521, United States

## Abstract

Interaction of nanoparticles (NP) with the lungs is an important field of study for controlling and understanding airborne nanotoxicity, as well as for advancing pulmonary drug delivery. In particular, NP interactions with lung surfactant (LS) films have been studied using experimental and computational means for particles of various physicochemical characteristics such as size, shape, and hydrophobicity. However, the dynamics of adhesion and encapsulation of heterogeneous NPs by biointerfaces remain poorly understood. In this work, we explore the effects of amphiphilic Janus NPs (JNPs) on lung surfactant films using dissipative particle dynamics (DPD) simulations. With a specially parametrized DPD model (Santo et al., *Colloids and Surfaces A*, 725, 137623 (2025)), we investigate the interfacial dynamics and interaction mechanisms of JNPs with model lung surfactant monolayers consisting of dipalmitoylphosphatidyl choline (DPPC) lipids. We find that the interaction of JNP with DPPC monolayers at a given surface density depends on the particle hydrophobic coverage and its initial orientation, resulting in three distinct scenarios: translocation, monolayer coating, and intercalation between the lipids as another amphiphilic entity. The surface pressure of the monolayer is found to increase nonmonotonically with JNP hydrophobic coverage, reaching a maximum at about 50% coverage. Interestingly, the surface pressure of a JNP–monolayer system is found to be similar to that of a pure monolayer at an effective area that excludes the JNP at the interface. Our simulation results elucidate the dynamics of JNP–lipid monolayer interactions and provide quantitative insights into how NPs of different surface chemistries could affect the functionality of LS films.

## Graphical abstract

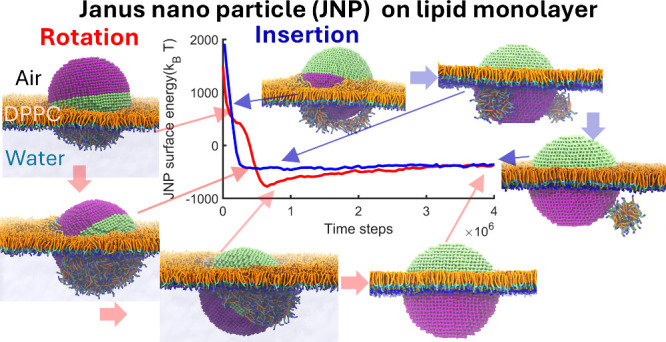

## Introduction

Nanoparticles (NPs) can enter the lungs by various means, including inhalation of air-borne particles or droplets as well as via NP-loaded pulmonary drug delivery.^1^ Upon deposition in the lungs, NPs interact with the lung surfactant (LS), potentially altering its functionality and affecting related biological processes.^2–5^ LS present at the air–water interface of the lung alveoli is a monolayer consisting mainly of phospholipids, cholesterol, and surfactant proteins: the major phospholipid component being the saturated dipalmitoylphosphatidyl choline (DPPC). LS regulates the surface tension of lung interfaces during contraction and expansion, which is essential to maintain the breathing process.^6^ While interactions of physicochemically different NPs with an LS monolayer have been characterized by experiments such as the Langmuir trough^7^ and constrained drop surfactometer (CDS),^8^ understanding nanoscale interfacial processes requires molecular simulations with quantitative accuracy, which has been difficult to achieve with currently available atomistic and mesoscale simulation methods. Here, we present a quantitatively consistent mesoscale dissipative particle dynamics (DPD) study of the interaction of Janus nanoparticles of different surface chemistries with model LS monolayers.

Being the major LS component, monolayers composed of DPPC lipids have long been used as the model LS film in experimental^7,9,10^ and computational studies.^11–15^ DPPC monolayers possess unique phase behavior upon compression and expansion, which is characterized by the surface-pressure area per lipid (P–A) isotherm. At low area per lipid, *a*_*L*_, or high surface lipid density, DPPC monolayers exhibit the liquid-condensed (LC) phase in which lipids are ordered and positioned upright along the surface normal. At lower lipid densities (high *a*_*L*_), monolayers exhibit the liquid expanded (LE) phase, in which lipids are randomly oriented without tail ordering. The LE-LC coexistence occurs at intermediate *a*_*L*_ values, which are characterized by a plateau region in the P–A isotherm. Experiments have shown that the exposure to NPs made of hydrophobic silica and TiO_2_,^9,10^ hydrophobic functionalized gold NPs,^16^ and CeO_2_ NPs^17^ affects the phase domain behavior and lipid packing and promotes disordered LE domains. The effects of NPs depend on their size (6–150 nm),^4,18^ shape, surface charge,^19,20^ and surface chemistry. NP incorporation at the interface leads to a reduction in effective area per lipid as well as the disruption of the monolayer structure at the interface, affecting the P–A Isotherm. A shift of the isotherm toward larger area per lipid has been observed.^7,16^ The mechanical and phase behaviors of DPPC monolayers are affected by the NP hydrophilicity and hydrophobicity; increasing the hydrophobicity has been reported to inhibit pulmonary surfactant function and cause particle retention in the monolayer.^8^

Since simulating nanometer scale solid particles with atomistic simulations is not practical, mesoscale simulation methods have been developed for studying processes on nanometer scales. Coarse-grained molecular dynamics (CGMD) simulations employing the MARTINI force field^21–24^ have been used extensively to study NP–LS interactions, which has been recently reviewed by Tang and Cui.^1^ Small (3–5 nm) hydrophobic NPs have been shown to induce structural disruptions in the monolayer with adhesion to the hydrophobic side and subsequent coating.^11^ The coating occurred only at low surface tension, whereas at high surface tension, the NPs remained embedded within the monolayer.^15^ Hydrophilic NPs of the same size, on the other hand, were found to pass through the monolayer without causing any disruption.^11,15^ However, larger hydrophilic NPs (12 nm) were found to translocate and drag lipids along, which form micelles in the water phase.^15^ CGMD simulations of Luo et al.^25^ have shown that NP shape becomes important for NPs of size larger than 5 nm. Several other studies also employed CGMD simulations to study shape effects on NP–LS interactions.^12–14^ Negatively charged NPs have been shown to associate with surfactant proteins (SPs), which are positively charged, while high charge on NPs affects their translocation due to adsorption on the lipid head groups.^26^

Janus NPs (JNPs) with both hydrophobic and hydrophilic surface regions can interact with both the water subphase and tail groups of the monolayer. Their amphiphilicity makes them unique, as the interfacial structural and mechanical properties of the monolayer can be drastically affected. Janus NPs have been shown to disrupt lipid bilayers,^27–29^ which suggests their potential applications in nanomedicine such as anticancer and antibacterial drug carriers. JNPs have been found to induce poration, protrusions, and collapse of giant unilamellar vesicles (GUVs).^28^ CGMD simulations have explored the mechanisms of JNP induced membrane disruption.^30^ Dissipative particle dynamics (DPD) is an alternative mesoscale simulation method used to efficiently model polymeric, colloidal, nanoparticle, and biomolecular systems.^31,32^ Ma and co-workers^33,34^ employed DPD simulations to explore JNP interactions with lipid membranes and showed that JNP incorporation into the bilayer depends on the initial orientation of adhesion. Depending on whether the JNP adheres with its hydrophilic or hydrophobic side facing the bilayer, distinct engulfment and insertion mechanisms were observed. These observations indicate that JNP–LS interactions involve more complex mechanisms compared to those involving NP with uniform surface chemistries. However, the effects of JNPs on LS monolayers have not been explored either experimentally or computationally, leaving a significant knowledge gap in understanding their impacts on lipid monolayers.

CGMD studies on NP–LS interactions predicted qualitative behaviors, as the main challenge utilizing atomistic and coarse-grained simulations for studying LS monolayers is the lack of quantitative accuracy of the force fields, especially when calculating the pressure–area isotherms. This is a consequence of the fact that most of the existing force fields, including the MARTINI force field, underestimate the air–water surface tension (72.8 mN/m at 293 K) and are therefore unable to quantitatively predict the P–A isotherms.^35,36^ The DPD method, in its standard form, cannot simulate gas–liquid interfaces.^37^ Wang et al. (WSN) have developed a simplified DPD approach to efficiently simulate gas–liquid interfaces with quantitative accuracy on the interfacial behavior.^38^ The authors applied the WSN method to study the temperature-dependent phase behavior of DPPC monolayers at the air–water interface.^35^ They developed a novel temperature scaling parameterization approach, wnich enabled calculating the DPPC pressure–area isotherms at different temperatures in quantitative agreement with experimental data. In the current work, we utilize the WSN model to study the interactions of JNPs with DPPC monolayers at the air–water interface at 293 K, thereby addressing the existing knowledge gap in the mechanistic understanding of JNP–LS interactions. We analyze the interfacial dynamics of single, isolated JNPs of different hydrophobic coverages and initial JNP orientations, governed by the surface energy of the JNP–monolayer system. We monitor the mechanical and morphological changes of the monolayer, quantitatively predicting the surface pressure of the JNP-loaded DPPC monolayer as a function of the particle hydrophobic coverage. The results provide novel physical insights into the interactions of JNPs with lung surfactant films.

## Methods

DPD is a coarse-grained simulation approach developed in the 1990s^37,39–41^ that has been extensively used for modeling soft material systems.^31,32^ DPD employs soft-core linear conservative forces (***F***_*ij*_^*C*^) as well as pairwise drag (***F***_*ij*_^*D*^) and random forces (***F***_*ij*_^*R*^) between CG beads to simulate their Newtonian motion with the effects of friction and thermal fluctuations,

$$F_{ij}^{C}\left(r_{ij}\right)=a_{ij}\left(1-\frac{r_{ij}}{R_{ij}}\right){\mathrm{r̂}}_{ij};F_{ij}^{D}\left(r_{ij}\right)=-\gamma w^{2}\left(r_{ij}\right)\left(v_{ij}.r_{ij}\right){\mathrm{r̂}}_{ij};F_{ij}^{R}\left(r_{ij}\right)=\sigma \theta_{ij}w\left(r_{ij}\right){\mathrm{r̂}}_{ij}$$ 1

Here, *a*_*ij*_ is the repulsion parameter, *R*_*ij*_ is the cutoff of the pairwise interaction, and γ is the friction coefficient. θ*_ij_* is a random noise function, σ^2^ = 2*k*_B_ *T*γ, and weight function *w* = 1 – *r*_*ij*_/*R*_*ij*_. This construct satisfies the fluctuation–dissipation theorem.^41^ The electrostatic interactions between ionic beads are taken into account using the *smeared-charge* approach.^42^ Bond lengths and bond angles are restrained at their respective equilibrium values by employing harmonic potentials. More details can be found from the extensive literature on DPD.^31,32^

### The Janus NP Model

We model JNPs as spherical particles of radius *R*_*NP*_ composed of DPD beads arranged in a cubic lattice and linked to their nearest neighbors via strong harmonic bonds. For improved computational efficiency, we choose the cubic lattice over the recommended close-packed lattices such as HCP, as the cubic lattice has a smaller number of beads and nearest neighbors. Therefore, the choice of the cubic lattice for modeling NPs leads to substantial reduction in the number of particles and bonds, enabling simulations of large NPs. We impose strong harmonic bonds between the nearest neighbor beads to ensure that NPs remain intact, keeping the radius of gyration *R*_*G*_ constant during the simulations (see Supporting Information (SI) Section 1). The JNPs are composed of hydrophilic K beads and hydrophobic L beads and characterized by their hydrophobic coverage, ϕ_*L*_, which is the ratio of the hydrophobic surface area to the total surface area of the particle. To create JNPs of different ϕ_*L*_, we make a spherical cap region of the NP hydrophobic (consisting of L beads), while the remaining region is hydrophilic (consisting of K beads). The hydrophobic coverage, ϕ_*L*_, depends on the height of the spherical cap, *h*,

$$\phi_{L}=\frac{A_{cap}}{A_{sphere}}=\frac{2\pi R_{NP}h}{4\pi R_{NP}^{2}}=\frac{h}{D_{NP}}$$ 2

where *D*_*NP*_ is the particle diameter. Figure 1a shows models of JNPs with different hydrophobic coverages.

**Figure 1:**
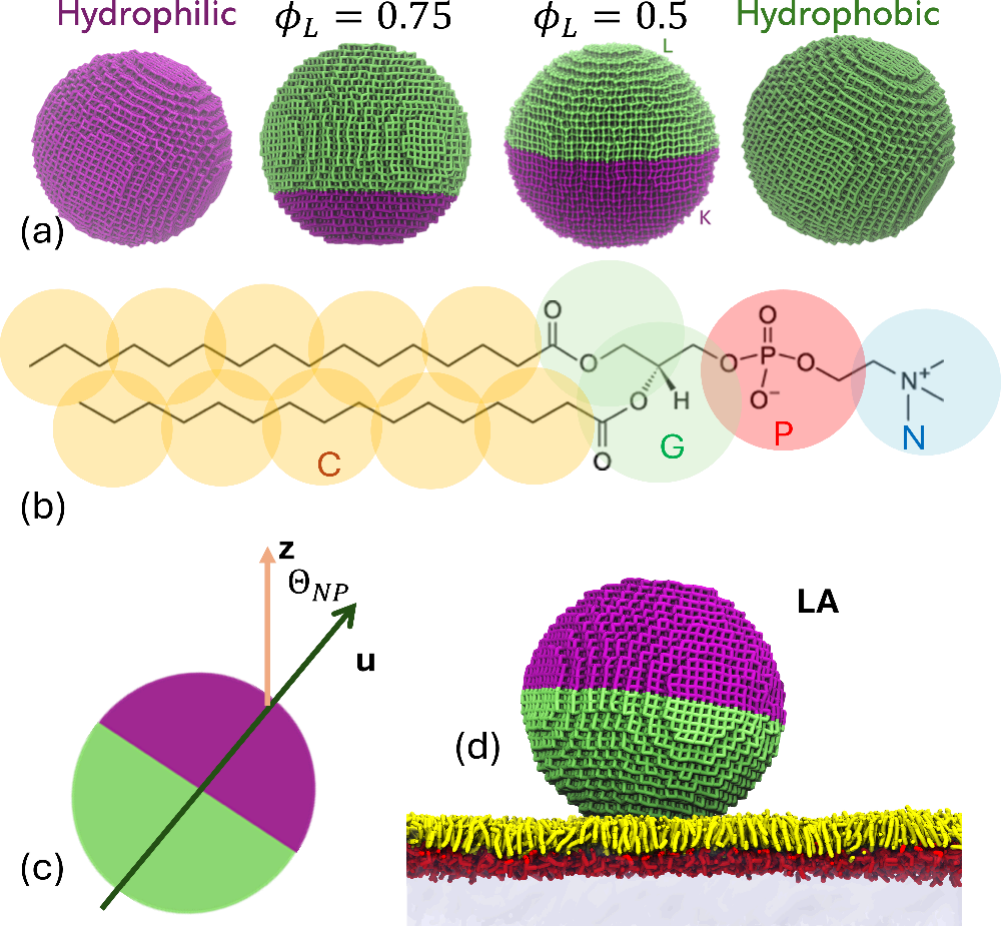
(a) Models of nanoparticles of 12 nm diameter and different hydrophobic coverages ϕ_L_. (b) The DPD model of the DPPC molecule. (c) Definition of the NP orientation vector **u** and the orientation angle Θ_NP_. (d) The initial configuration of the JNP–monolayer system in lipophile adhesion (LA) mode. Nanoparticle colors: purple - hydrophilic, lime - hydrophobic. Monolayer colors: yellow - lipid tails, red - lipid headgroup. Water subphase is colored light blue.

### System Setup and Initial Configurations

We use the previously parametrized WSN model^35^ of DPPC monolayers at the air–water interface. In this model, water is taken as the reference compound with the CG water bead (W) representing 3 water molecules. The water bead size *R*_*WW*_ = *R*_*c*_ = 0.646 nm is taken as the DPD unit length. The bead number density is ρ_*W*_ = 3*R*_*c*_^–3^ and *a*_*WW*_ = 25.0*k*_*B*_ *T*/*R*_*c*_. The DPD pressure of 23.7 corresponding to this water system is taken as the reference pressure. The DPPC molecule is modeled as composed of 14 beads representing the choline (N), the phosphate (P), the glycerol backbone (G), and the alkyl groups in the tails (C) (Figure 1b). The gas phase is modeled as consisting of fictitious beads (B) according to the original WSN model.^38^ B-beads interact with other beads with an exponential conservative force,

$$F_{ij}^{\exp}\left(r_{ij}\right)=a_{Bj}\frac{e^{b_{Bj}r_{ij}/R_{Bj}}-e^{b_{Bj}}}{1-e^{b_{Bj}}}{\mathrm{r̂}}_{ij}$$ 3

where *a*_*Bj*_, *b*_*BJ*_, and *R*_*BJ*_ are parameters determined through matching the interfacial properties of water and the monolayer. The exponential force, eq 3, upon particle contact, increases more steeply than the linear conservative force, eq 1, mimicking a hardcore interaction. This parametrization is shown to be capable of modeling the interfacial dynamics with accurately reproduced monolayer surface tension. The repulsion parameters of JNP beads are chosen similar to water (for K) and tail beads (for L), that were systematically parametrized.^35^ All parameters are provided and detailed in the Supporting Information (Section S2).

JNPs are relaxed in water by a short DPD simulation for 50000 steps. We simulate the interactions of single JNPs with a DPPC monolayer, assuming a low NP concentration at which JNPs remain isolated without aggregation. A pure DPPC monolayer consisting of 1400 DPPC lipids is equilibrated at area per lipid *a*_*L*_ = 0.6 nm^2^ for 2 million steps by simulating a double monolayer system.^35^ We chose this area per lipid because it is close to the LC transition point and therefore allows more pronounced JNP effects to be revealed. JNPs adhere from the air phase to the hydrophobic side of each monolayer. The simulation system consists of a double monolayer–JNP system with a water slab in the *X*–*Y* plane in contact with the air phase above and below in the *Z*-direction (see SI Figure S2). Both air–water interfaces are covered by DPPC monolayers, which makes the system periodically symmetric in the normal *Z*-direction. The initial configurations of JNP adhering on the hydrophobic side of the DPPC monolayer are constructed using the Packmol^43^ program with pre-equilibrated DPPC monolayers and JNPs.

Due to the anisotropic surface chemistry of the JNPs, the orientation of JNP, when it comes into contact with the monolayer, influences its interfacial dynamics. For instance, JNPs can adhere to the monolayer with its hydrophobic side, or hydrophilic side, or both facing the monolayer. It may be argued that since the monolayer is hydrophobic, JNPs may tend to adhere to its hydrophobic side. However, when NPs at random orientations approach the monolayer, the hydrophobicity is felt only over short distances. The much slower NP dynamics may not allow rotation of the flip sides to adhere with the NP hydrophobic side. Therefore, we consider two typical cases of NP adhesion on the monolayer: the lipophile adhesion (LA) in which the JNP adheres to the monolayer facing with its hydrophobic side, (Figure 1d) and the hydrophile adhesion (HA) in which it adheres with its hydrophilic side. The adhesion at random orientations may be understood in terms of the extreme cases. The JNP orientation with respect to the monolayer is characterized by the orientation angle Θ*_NP_*, which is the angle between the monolayer normal and the JNP director ***u*** defined by,

$$u=\frac{R_{com}^{K}-R_{com}^{L}}{\left|R_{com}^{K}-R_{com}^{L}\right|}$$ 4

where ***R***_*com*_^*K*^ and ***R***_*com*_^*L*^ are the centers of mass of the hydrophilic and hydrophobic caps, respectively, which lie on the axis of the sphere. Thus, ***u*** is an axial unit vector directed from the hydrophobic cap to the hydrophilic cap (Figure 1c). The orientation angle is then given by,

$$\Theta_{NP}=\arccos\left(u.ẑ\right)$$ 5

Note that the adhesion modes, LA and HA, correspond to Θ*_NP_* ∼ 0 and Θ*_NP_* ∼ 180°, respectively.

### Simulation Details

DPD simulations are performed using the DL_MESO software,^44^ in the LA and HA modes of adhesion with JNPs of different hydrophobic coverages in the range of 0.0–1.0. ϕ_*L*_ values of 0 and 1 represent hydrophilic and hydrophobic NPs, respectively. All simulations are run at *a*_*L*_ = 0.6 nm^2^, with a lateral size *L*_*x*_ = *L*_*y*_ ≈ 44.6*R*_*c*_ and a normal dimension of *L*_*z*_ ≈ 80–90*R*_*c*_ with a total number of particles of ≈486000–546000. Periodic boundary conditions are used in all directions. The initial configuration is equilibrated during a short 20000 step NPT simulations with large mass (∼50 times) imposed on the NP and DPPC beads, in order to make them immobile and to allow water and gas subphases to equilibrate. Following this, a 50000 step NPT simulation is performed with actual masses (see Table S2) to relax the system at pressure *P* = 23.7. The temperature is set at *T*_*DPD*_ = 0.65, which corresponds to a real temperature *T*_*real*_ = 293 K, following the temperature scaling approach developed in our previous work.^35^ Subsequently, an NVT simulation is performed for 2–4 million steps until the average surface tension of the monolayer is constant and equilibrated. Details of the simulations are provided in the Supporting Information (Section S3). The time evolution of the systems is monitored during the second NPT equilibration and NVT simulation. The surface tension of the monolayer calculated by,

$$\gamma_{m}=\frac{L_{z}}{2}\left(P_{zz}-\frac{P_{xx}+P_{yy}}{2}\right)$$ 6

where *P*_*xx*_ and *P*_*yy*_ are lateral pressure tensor components and *P*_*zz*_ is the pressure along the normal *z*-direction. The surface pressure of the monolayer is defined as,

$$\Pi =\gamma_{0}-\gamma_{m}$$ 7

where γ_0_ is the air–water surface tension.

## Results and Discussion

### JNP Interfacial Behavior

The interfacial dynamics of JNPs with different hydrophobic coverages in LA and HA modes, obtained from DPD simulations, are depicted in Figure 2. Hydrophilic NPs, upon contact with the monolayer, quickly attract water beads below and pierce through the monolayer, eventually translocating to the water subphase completely (Figure 2A). The NP tears off the monolayer, which eventually heals itself once the translocation is complete. In the water phase, the NP tends to adhere to the lipid head groups and therefore remains right below the interface. Hydrophobic NPs, on the other hand, adsorb on the monolayer at the hydrophobic side, half-coated by the monolayer. The extent of this coating could depend on the surface lipid density and the surface tension of the interface. These results are consistent with the available experimental observations^8,10^ and CGMD simulations results.^11,15^ Valle et al.^8^ have reported that the NP retention in LS monolayers increases with hydrophobicity of the NP. Coarse-grained simulations^11^ have reported translocation of hydrophilic NPs as well as surface pressure-dependent monolayer disruption by hydrophobic NPs.^15^ Our DPD model has quantitatively reproduced air–water surface tension as well as DPPC P–A isotherm at 293 K.^35^ Therefore, unlike MARTINI simulations, our simulations have the correct surface tension, which is the crucial factor determining the NP interfacial behavior and are expected to be quantitatively accurate.

**Figure 2:**
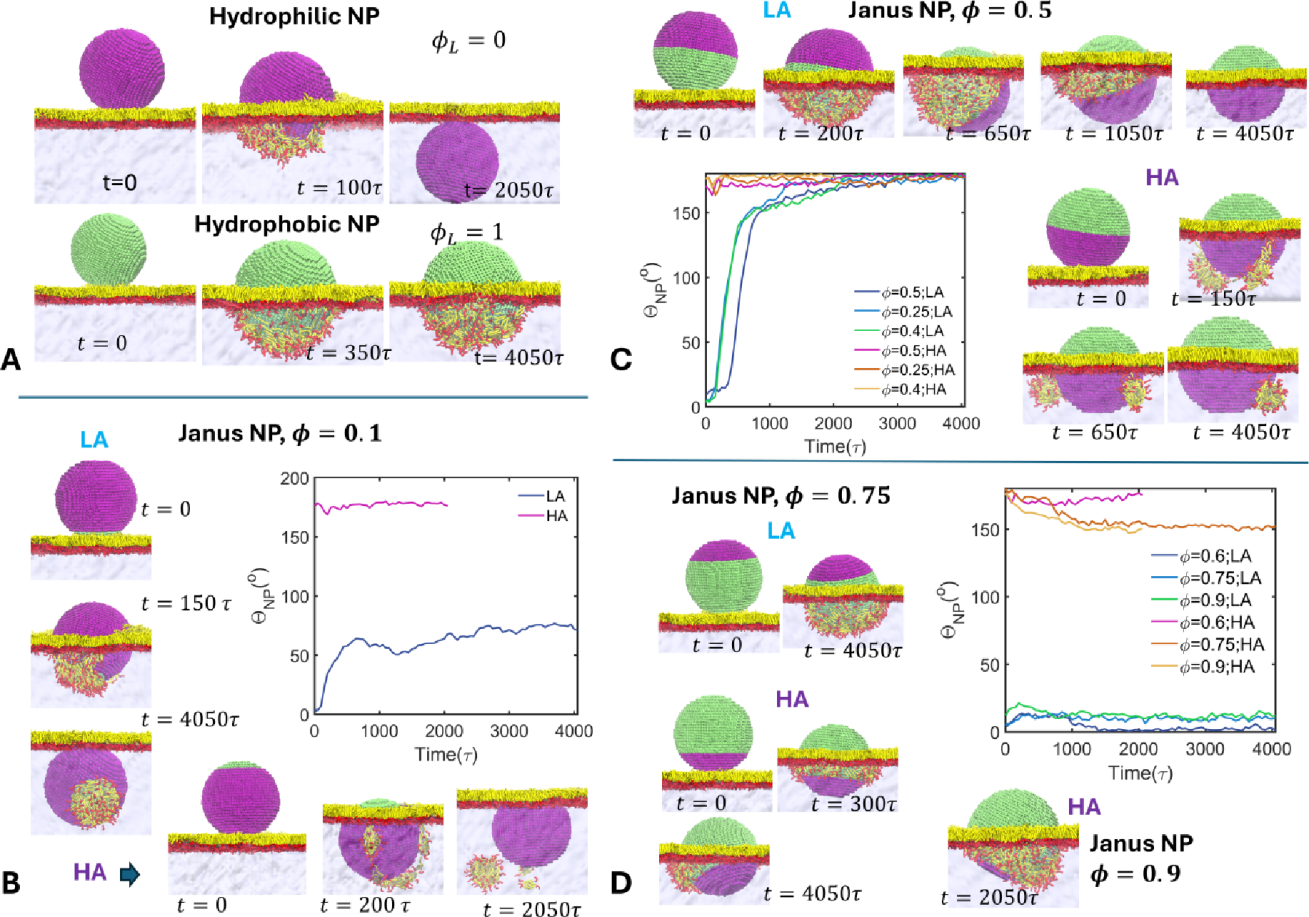
Interfacial dynamics of the JNPs of different hydrophobic coverages in lipophile and hydrophile adhesion modes. (A) Interaction of purely hydrophilic and purely hydrophobic NPs with the DPPC monolayer. (B–D) JNP–monolayer interactions in LA and HA modes at low, intermediate, and high hydrophobic coverages, respectively. The variation of the NP orientation angle Θ_NP_ as a function of time for each case is also provided. Color scheme is the same as that of Figure 1.

JNPs with very low hydrophobic coverage, ϕ_*L*_ = 0.1, are also found to translocate to the water subphase in LA mode (Figure 2B). In this case, as the JNP adheres to the film, it gets coated by the lipid monolayer on its hydrophobic area, which brings its hydrophilic part into contact with water. Subsequently, the NP rotates (by angle Θ*_NP_* ∼ 60°) to completely solvate its hydrophilic part. However, this process tears off the monolayer, dragging the lipids that coat the NP’s hydrophobic surface into the water, resulting in a loss of lipids from the monolayer. In the HA mode, the JNP inserts directly into the water phase without rotation, disrupting the monolayer and causing a loss of lipids that form micelles in the water phase. Here, JNP occupies the interface with its hydrophobic part in contact with lipid tails.

At intermediate hydrophobic coverages (ϕ_*L*_ = 0.25–0.5), the LA mode initially leads to coating of the NP’s hydrophobic surface by monolayer lipids, followed by a large-angle rotation that exposes the NP’s hydrophilic side to the water subphase (Figure 2C). Flipping of the NP, driven by solvation by water, occurs rather abruptly and temporarily stabilizes at an angle Θ_*NP*_^*m*^ in the range of 115–140°, which depends on the hydrophobic coverage. Subsequently, the NP undergoes a slower rotation and eventually attains a configuration in which the NP intercalates at the interface, with an orientation angle of 180°, compressing the lipids in the monolayer without any damage. In the HA mode, the JNP pierces and inserts through the monolayer without rotation, as before, disrupting the monolayer and causing lipid loss into the water subphase.

The simulations show that JNP intercalation at the monolayer–water interface occurs through distinct mechanisms in LA and HA modes. In the LA mode, intercalation proceeds gradually via NP rotation without lipid loss, whereas in the HA mode it occurs by direct insertion, resulting in monolayer disruption and lipid loss. The lipid loss observed in our simulations may be transient and limited by the simulation time scales. We observe that some lipids that moved into the water phase returned to the monolayer, while others did not. Once detached, the lipids form micelles or other morphologies and become stabilized in the water phase. As shown in Figure S4, one of the two micelles formed after NP insertion in the HA mode at ϕ_*L*_ = 0.5 returns to the monolayer, but the other remains in the bulk until the end of the simulations. The reassociation of the micelles with the monolayer may be influenced by several factors such as slower micellar diffusion, surface pressure of the monolayer, and interactions with other entities in the water subphase, such as lipid reservoirs beneath the lung surfactant layer, which may lead to much longer reassociation times or even permanent damage.

At high hydrophobic coverages (ϕ_*L*_ > 0.5), JNPs remain adsorbed on the hydrophobic side of the monolayer in the LA mode, coated by lipids, without water contact (Figure 2D). In this configuration, the JNP cannot rotate because its hydrophilic region is located far from the interface. In the HA mode, however, the JNPs can still intercalate via an insertion mechanism. This intercalation is accompanied by partial coating of the hydrophobic side by the monolayer and tilting of the NP, which leads to a locally curved monolayer–water interface.

We conclude that the interaction of JNPs with DPPC monolayers at the air–water interface can lead to three distinct outcomes: (1) translocation, (2) intercalation, and (3) monolayer coating. These interfacial adhesion and translocation processes can disrupt the monolayer, cause lipid loss, and alter its mechanical properties. These results add to the critical knowledge gap by providing quantitative characterization of JNP interfacial behavior on lipid monolayers.

### Surface Energy of the JNP–Monolayer System

The JNP interaction with DPPC monolayers at the air–water interface is governed by the change in the interfacial energy upon JNP adhesion. The equilibrium configuration of the NP at the interface is determined by the minimum of the surface energy, which is the sum of the NP surface adsorption energy (*E*_*NP*_) and the interfacial energy of the monolayer region that is not in contact with the NP ( ${E_{m}}^{\prime }$ ). Thus,

$$E_{S}=E_{NP}+E_{m}^{\prime }$$ 8

where the prime superscript indicates that the area occupying the NP is excluded. The monolayer free energy is given by the surface tension of the monolayer (γ_*m*_),

$$E_{m}^{\prime }=\gamma_{m}\left(L_{x}L_{y}-A_{I}^{NP}\right)$$ 9

where *A*_*I*_^*NP*^ is the interfacial area occupied by the NP.

To calculate the surface energy of the JNPs, we consider the relative repulsion and attraction between NP hydrophilic and hydrophobic beads, K and L, and the other beads in the system. Since DPD pairwise conservative potential, *V*_*ij*_, is always repulsive, it is the relative interbead repulsion that determines an effective attraction or repulsion between the beads. For instance, the hydrophilic NP beads K attract water beads since the DPD repulsion between K and W beads (*a*_*KW*_) is smaller than intracomponent W–W (*a*_*WW*_) and K–K (*a*_*KK*_) repulsions. The bead–bead contact energy between an NP bead and any other bead is determined as,

$$\epsilon_{\alpha j}=\frac{\int_{0}^{R_{\alpha j}}\Delta V_{\alpha j}\left(r\right)g_{\alpha j}\left(r\right)r^{2}dr}{\int_{0}^{R_{\alpha j}}g_{\alpha j}\left(r\right)r^{2}dr}$$ 10

where *r* is the interbead distance and *g*_α*j*_(*r*) is the radial distribution function. α = K, L corresponds to the NP bead index, and *j* = N, P, G, C, W, B denote lipid, water, and gas beads. Δ*V*_*ij*_ is the mismatch potential,

$$\Delta V_{\alpha j}=V_{\alpha j}-\frac{1}{2}\left(V_{\alpha \alpha }+V_{ij}\right)$$ 11

For beads with standard DPD interactions,

$$V_{\alpha j}\left(r\right)=\frac{1}{2}a_{\alpha j}R_{\alpha j}{\left(1-\frac{r}{R_{\alpha j}}\right)}^{2}$$ 12

The interactions with gas beads B are governed by the exponential potential,

$$V_{Bj}\left(r\right)=\frac{a_{Bj}}{1-e^{b_{Bj}}}\left(\left(r-R_{Bj}\right)e^{b_{BJ}}+\frac{R_{Bj}}{b_{Bj}}\left(e^{b_{BJ}}-e^{b_{BJ}r/R_{Bj}}\right)\right)$$ 13

(see SI Section S4.2). The surface energy of the NP is then calculated by knowing the number of beads in contact with the NP beads at the surface, *n*_α*j*_ (α = K, L)

$$E_{S}=E_{NP}^{K}+E_{NP}^{L}=\underset{j}{\sum }\left(\epsilon_{Kj}n_{Kj}+\epsilon_{Lj}n_{Lj}\right)$$ 14

To calculate the bead–bead contact energy, eq 10, we determine the radial distribution functions *g*_α*j*_(*r*) for each respective bead pair from the DPD simulations of free NP beads, K or L, in bulk environments consisting of the other bead type (W, B, N,G, P, and C) (see SI Section S4.1). The calculated energies are provided in Table S5, which shows that ϵ_α*j*_ values are of the order of a fraction of *k*_B_ *T*. As expected, favorable bead–bead interactions have negative energy, while unfavorable interactions have positive energy. Typical examples are ϵ_*KW*_ = −0.173*k*_B_ *T*, ϵ_*KC*_ = 0.426*k*_B*T*_, ϵ_*LW*_ = 0.429*k*_B_ *T*, and ϵ_*LC*_ = −0.111*k*_B_ *T*.

Figure 3 depicts the NP interfacial dynamics in terms of the change in the surface energy over time. As shown in Figure 3a, the surface energy of the JNP with ϕ_*L*_ = 0.5 in the LA mode decreases steeply as the NP adheres to and becomes coated by the monolayer. *E*_*NP*_ reaches a plateau once the monolayer completely coats the NP surface that is hydrophobic. At this point, the NP would be completely stabilized if the hydrophobic coverage is high (ϕ_*L*_ > 0.5) as inferred from Figure 3b. At an intermediate hydrophobic coverage, the monolayer-coated NP becomes unstable; as it rotates, its hydrophilic region becomes solvated by water, leading to a sharp decrease in the surface energy, which reaches a minimum. This minimum, which occurs at an orientation angle Θ_*NP*_^*m*^ = 115–140° corresponds to a tilted NP configuration, as shown in Figure 3a. This configuration is an energy minimum for the NP because it has substantial monolayer coverage and solvation by water. Any further rotation will reduce monolayer coverage and thereby increase the NP surface energy.

**Figure 3:**
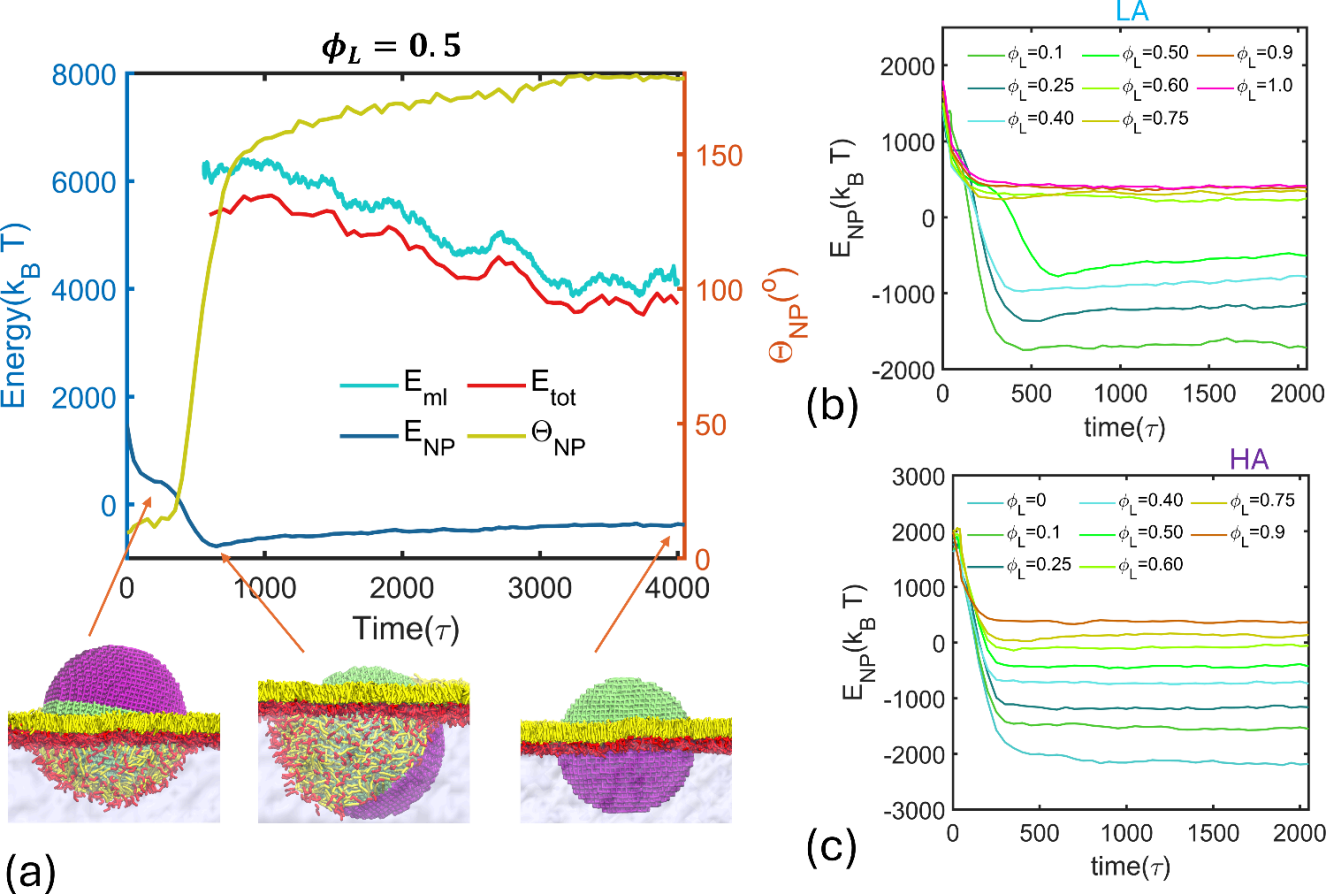
(a) The variation of surface energy of the JNP–monolayer system at 50% hydrophobic coverage during the simulation in the lipophile adhesion mode. The snapshots of the representative NP states at the interface of monolayer coverage, minimum NP surface energy, and intercalation are given. (b) and (c) provides the surface energy of JNPs of different hydrophobic coverages as a function of time in the LA and HA modes, respectively.

Upon reaching the *E*_*NP*_ minimum, JNP continues to rotate although at a much slower rate with its surface energy increasing, as indicated by the variation of Θ*_NP_* with time in Figure 3a. The NP eventually attains an intercalated configuration, adopting a parallel orientation (Θ*_NP_* = 180°) of its hydrophilic/hydrophobic caps with respect to the interface. This occurs despite the increase in NP energy because the NP energy minimum does not correspond to the equilibrium state of the combined monolayer–NP system. As shown in Figure 3a, the monolayer energy and the total energy of the monolayer–NP system continue to decrease, reaching a minimum at the final intercalated NP configuration. The monolayer energy decreases as the NP rotates as more and more lipids occupy the interface, which reduces the surface tension. Note that this behavior depends on the lipid density; the NP may remain in the tilted configuration at lower area-per-lipid values, where further reduction in surface tension is not possible with the addition of lipids. In the HA mode, the JNP directly attains the minimum-energy configuration through the insertion mechanism (Figure 3c). In this case, the energy minimum of the monolayer–NP system also depends on the extent of monolayer damage and lipid loss.

### Effects of JNPs on Monolayer Surface Pressure and Phase Behavior

The initial monolayer at *a*_*L*_ = 0.6 nm^2^ exhibits an LE-LC coexistence, with a surface pressure of ∼8 mN/m that falls well within the plateau region of the surface pressure–area curve of DPPC monolayers.^35^ To analyze the effects of JNP adhesion on the 2D phase behavior of the monolayers, we calculate the lipid tail order parameter *S*,

$$S=\frac{1}{2}\left(3\cos^{2}\theta -1\right)$$ 15

where θ is the angle between the tail vector and the monolayer normal along z, as defined in our previous work.^35^ Figure 4 (and Figure S5) depicts the top-view snapshots of the monolayers at the end of the simulations for various JNP hydrophobic coverages and adhesion modes, with lipids colored according to their tail order parameters, averaged over the two hydrocarbon tails. The LC phase at high order with tails upright is distinguished with a red region, while the LE phase at a lower order has tails colored green or blue. At ϕ_*L*_ = 0, the purely hydrophilic NP translocates across the interface, and the monolayer structure and phase behavior remain unchanged. At 10% hydrophobic coverage in the LA mode, the NP translocates while carrying adsorbed lipids, leading to an increase in the liquid-expanded (LE) phase. The JNP intercalation at the interface leads to an increase in the LC phase since, the area available to lipids, the residual area *A* ^*res*^ = *L*_*x*_ *L*_*y*_ – *A*_*I*_^*NP*^ decreases. We estimate the number of lipids in the LC phase per residual area as,

$$f^{LC}=\frac{n_{L}\left(S>0.6\right)}{A^{res}}$$ 16

considering *S* > 0.6 to be the LC phase. As shown in Figure S6 and inferred from Figure 5, *f* ^LC^ increases with hydrophobic coverage up to ϕ_*L*_ = 0.5, where JNP intercalation occurs in both LA and HA modes. For ϕ_*L*_ > 0.5, *f* ^LC^ drops close to its pure-monolayer value in the LA mode due to monolayer adhesion, whereas in the HA mode, it decreases more gradually because intercalation still occurs. Note that we do not observe any increase in LE phase due to monolayer adhesion at high hydrophobic coverages. However, an increase in LE phase can occur due to extensive lipid loss from the monolayers as in the LA mode at 10% hydrophobic coverage.

**Figure 4:**
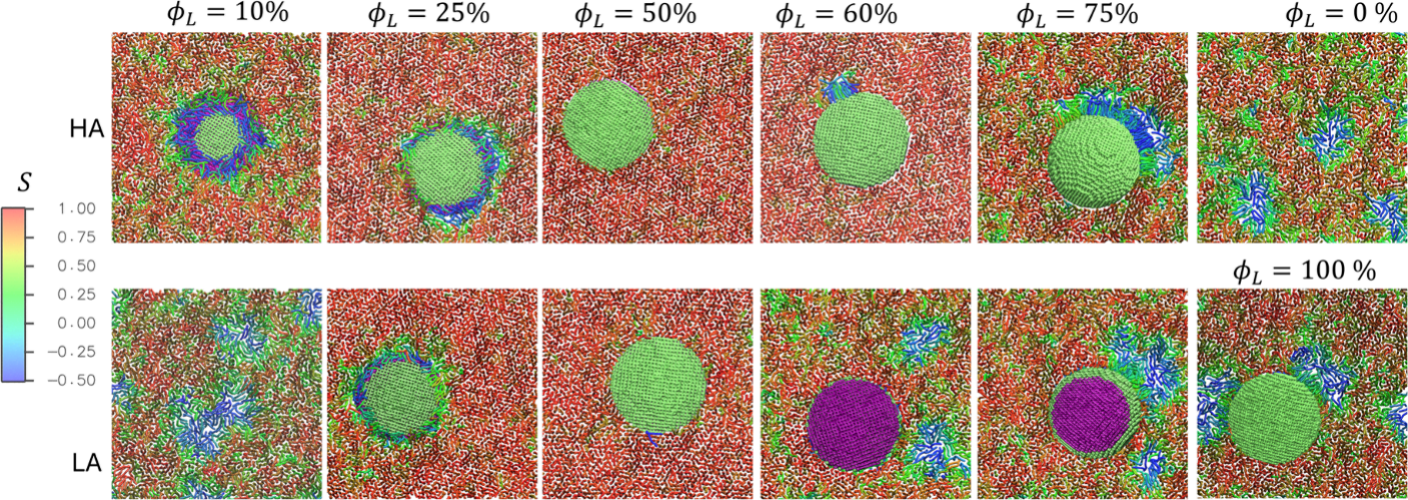
Effects of JNPs on the two-dimensional phase behavior of DPPC monolayers. Top-view snapshots of the monolayers at the end of the simulations for different hydrophobic coverages are shown. Lipids are colored according to tail order, distinguishing the LC (red; high order) and LE (blue; lower order) phases. The JNP is colored with the hydrophobic region in lime and the hydrophilic region in purple.

**Figure 5:**
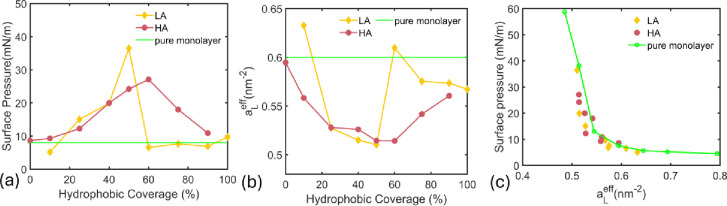
(a) The surface pressure and (b) the effective area per lipid of the monolayer at different hydrophobic coverages. (c) The monolayer surface pressure as a function of the effective area per lipid.

The variation of the monolayer surface pressure, Π, with hydrophobic coverage in LA and HA modes is shown in Figure 5. The surface pressure is found to increase with JNP amphiphilicity, peaking at around 50–60% hydrophobic coverage-maximum amphiphilicity. Both hydrophilic and hydrophobic NPs are found to exhibit minimal effects on the surface pressure. The effects of JNPs on Π can be analyzed based on the three scenarios—translocation, intercalation, and monolayer adhesion. Being a function of the area per lipid of the monolayer, Π is unchanged when JNPs translocate without damaging the monolayer. Therefore, purely hydrophilic NPs may not have any effect on the monolayer surface pressure (Figure 5a), as long as they do not cause any damage such as loss of lipids. Intercalation of JNPs at the interface and/or the loss of lipids affect the surface pressure, as both processes alter the available area for the lipids and the LE–LC phase behavior; the increase in LC phase due to intercalation leads to an increase in surface pressure. The effects of JNPs on the monolayer surface pressure can be analyzed in terms of an effective area per lipid of the monolayer after JNP adhesion. The effective area per lipid after interaction with JNP is given by the residual area of the monolayer and the residual number of lipids at the interface, *n*_*L*_^*res*^

$$a_{L}^{eff}=\frac{A^{res}}{n_{L}^{res}}=\frac{L_{x}L_{y}-A_{I}^{NP}}{\left(n_{L}-n_{L}^{w}\right)}$$ 17

where *n*_*L*_^*w*^ is the number of lipids that have moved to the water subphase or coated on the JNP in the case of monolayer adhesion.

Figure 5b shows that *a*_*L*_^*eff*^ varies differently in LA and HA modes due to the difference in the mechanisms of the JNP interfacial dynamics. At low hydrophobic coverage, the loss of lipids increases *a*_*L*_^*eff*^ and decreases Π in the LA mode, while at intermediate hydrophobic coverages, the effective area per lipid dips due to the JNP exclusion, increasing the LC phase and the surface pressure. At high ϕ_*L*_, monolayer coating of the NP affects *a*_*L*_^*eff*^; the monolayer seems slightly compressed as the density of the coated lipids on the NP surface is lower. The lipids tend to strongly stick to the NP surface with all tail beads adsorbed on the NP surface, leading to a lower lipid density compared to that of the monolayer. Figure 5c suggests that the surface pressure of the JNP–monolayer system is similar to that of a pure monolayer of the corresponding effective area per lipid in both HA and LA modes. This result, further supported by the observed LE–LC phase behavior shown in Figure 4, is particularly interesting because it is challenging to access experimentally and no prior simulations have demonstrated it with quantitative consistency.

Overall, our results provide quantitative predictions of the mechanisms governing the JNP interfacial behavior on lipid monolayers. Note that the JNP interfacial behavior also depends on surface pressure. For example, at high surface pressures, nanoparticles may be stabilized at their surface-energy minimum (Figure 3a) without completely flipping, as further rotation would increase the surface pressure. Monolayer coverage on hydrophobic nanoparticles is also surface-pressure-dependent; at sufficiently high surface pressures, lipids may fully cover the nanoparticle, leading to encapsulation. Another important factor is nanoparticle size, as the JNP interfacial behavior is strongly influenced by nanoparticle curvature. For instance, rotation and flipping may become increasingly difficult for smaller nanoparticles, whereas the insertion of larger nanoparticles may lead to significant monolayer damage. These effects will be analyzed in future work.

## Conclusions

Understanding the dynamics and fate of nanoparticulate matter in the lungs is important for controlling environmental pollution, nanotoxicity, and pulmonary drug delivery. Experimental studies on NP interactions with model lung surfactant films are not very conclusive; understanding nanoscale behavior of biointerfaces is beyond the reach of experimental methods and calls for molecular simulations. However, quantitatively accurate molecular simulations of biointerfaces are difficult to perform with the available computational approaches. Notably, the interactions of amphiphilic Janus nanoparticles with lung surfactant films remain unexplored, both computationally and experimentally. With our recently developed DPD model that quantitatively reproduced the interfacial behavior of model lung surfactant films, we have investigated the JNP interactions with DPPC monolayers.

Our DPD simulations performed at 293 K show that purely hydrophilic NPs translocate across DPPC monolayers without altering the interfacial structure and surface tension, whereas purely hydrophobic NPs are retained on the film’s hydrophobic side, partially coated by the monolayer, and thereby disrupting the interfacial structure. These results are consistent with the existing experimental and CGMD simulation results. However, amphiphilic NPs exhibit different behaviors depending on the hydrophobic coverage and the initial JNP orientation. We consider two typical cases of JNP orientation: the lipophile adhesion when the JNP contacts the monolayer with its hydrophobic side and the hydrophile adhesion when the JNP contacts the monolayer with its hydrophilic side. In general, JNPs tend to intercalate into the monolayer as another amphiphilic entity between the lipids, which decreases the effective area per lipid due to the excluded volume effect.

While JNPs of low hydrophobic coverages can translocate across the DPPC monolayer, intercalation is the main mechanism of interfacial effects of JNPs of up to 50% hydrophobic coverage. In the LA mode, the JNP first gets coated with the monolayer and subsequently rotates to complete the intercalation, while in the HA mode, the JNP directly inserts into the monolayer. So, it can be concluded that JNPs with a random initial orientation will adopt a combination of the rotation and insertion mechanisms. We also find that the insertion mechanism mostly leads to a loss of lipids from the monolayer. At high hydrophobic coverages, JNPs are either retained on the monolayer hydrophobic side partially coated by the monolayer or intercalated with partial coating, depending on the initial mode of adhesion. The simulations suggest three scenarios for the JNP–monolayer interaction: translocation, intercalation, and monolayer coating.

We show that the interfacial dynamics of JNPs are governed by the surface energy of the monolayer–JNP system. While the JNP energy is minimized due to solvation of the hydrophilic side and the coating of the hydrophobic side with lipid tails, monolayer equilibrium is controlled by a reduction in the surface tension. The surface pressure of the monolayer is found to vary nonmonotonically with JNP hydrophobic coverage, with a maximum increase with respect to the surface pressure of the pure monolayer achieved at 50–60%. Interestingly, the surface pressure of the JNP–monolayer system is found to be similar to that of the pure monolayer at an effective area per lipid, which although expected, has not been shown through simulations with quantitative accuracy.

In this work, we have considered the simplest system of JNPs of different hydrophobicity yet only one size (12 nm) interacting with a one-component (DPPC) lipid monolayer. Additional limitations of our approach include the restricted system size and accessible time scales, which prevent the observation of large-scale spatial and temporal phase-domain behavior and may influence certain predictions, such as those shown in Figure 5c. The proposed DPD model can be extended to more complex nanoparticle–lipid monolayer systems composed of different types of lipids and containing cholesterol and surfactant proteins. A multicomponent environment can significantly affect the mechanisms of JNP interactions, as the presence of cholesterol and surfactant proteins has been shown to influence monolayer phase behavior and structural properties in the presence of NPs.^45,46^ The next steps are to perform an extended study of the size dependence of nanoparticle adhesion and perform simulation at iso-stress conditions. It is interesting to consider the mechanisms of aggregation of NPs adhered to lipid monolayers, considering larger systems with two or more particles in the simulation cell. Most importantly, the simulation results must be compared with specially designed experiments to secure similar conditions in modeling and experimentation. Such experiments are on their way, and the comparison will be presented in a follow-up paper.

## Supplementary Material



## Notes

Details of the simulation system, DPD parameters, JNP modeling, and calculation of surface energies (PDF)
