# Supplementary material for: Interactions of Amphiphilic Janus Nanoparticles with Lipid Monolayers
Source: Langmuir. 2026 Feb 12;42(7):5676–85. doi: 10.1021/acs.langmuir.5c06024 (PMC12937096; doi:10.1021/acs.langmuir.5c06024)
Supplement: Supplementary file 1 [file la5c06024_si_001.pdf]

Supporting Information

Interactions of amphiphilic Janus nanoparticles with lipid monolayers

Kolattukudy P. Santo[1], Younjin Min[2], and Alexander V. Neimark[*,1]

[1]*Department of Chemical and Biochemical Engineering, Rutgers, The State University of New Jersey, Piscataway, NJ, USA, 08854*

[2]*Department of Chemical and Environmental Engineering, University of California Riverside, Riverside, CA, USA, 92521*

[*]Corresponding author: aneimark@rutgers,edu

Table of Contents

S1. Nanoparticle model 2

S2. DPD interaction parameters 2

S3. The JNP-DPPC monolayer simulation set up 4

S4. Surface Energy Calculations 5

S4.1 The radial distribution functions 5

S4.2 Potential energy of the exponential interaction 6

S5. JNP-induced Morphological Behavior 7

S5.1 Monolayer disruption and healing 7

S5.2 Monolayer phase behavior 7

References 8

S1. Nanoparticle model

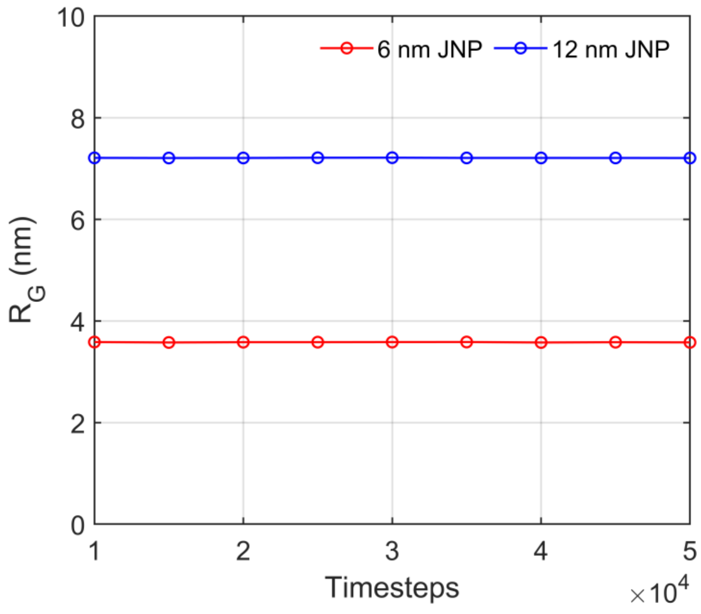

Figure S1. Radius of gyration of the JNPs of different sizes as a function of time during DPD simulations.

JNPs are modelled as spherical particles composed of DPD beads arranged in a cubic lattice. Strong harmonic potentials are applied to hold the NP beads together to maintain the spherical shape of the NPs. Radius of gyration R_G is a quantity that characterizes the shape of an object, as shape changes results in change in R_G.

To ensure the NPs are intact, DPD simulations are performed with a single NP in water. A $30 \times 30 \times 30\ R_c^3$ system is created with JNP and water beads packed to a density of $\rho = 3\ R_c^{-3}$. NPT simulations at temperature $k_BT = 1$ and pressure 23.7 are run for 50000 steps. During the simulations, NP remained intact as R_G remained practically constant (Figure S1) in the case of two NPs of diameter 6 nm and 12 nm.

S2. DPD interaction parameters

The DPD interactions between gas, water and lipid beads were parametrized systematically in our previous work at a temperature $T_{DPD} = 0.65$ that corresponds to 293 K.[1] The parameters of the NP beads- K and L- are chosen to mimic their hydrophilicity and hydrophobicity similar to water and tail beads and no specific parametrization was done. The interactions between the NP beads are chosen slightly repulsive, which helps in holding the NP shape intact. There are no specific NP-NP interactions considered in this work. The repulsion parameters between the water, lipid and the NP beads are provided in Table S1. The relative mass, charge and bonded interactions parameters are provided in Table S2. The NP beads have masses same as that of water ($m_K = m_L = 1.0$) and carries no charge. The nearest neighbor bonds between the NP beads are held by harmonic potentials with bond length $l_b = 0.65\ R_c$ and force constant $k_b = 600.0\ k_BT/R_c^2$. Gas-NP interactions are set the same as water and tail beads for K beads and L beads respectively.

Table S1. The DPD repulsion parameters (a_{IJ}) and bead sizes (R_{IJ}) of water, lipid and NP beads/

$a_{IJ}(k_BT)$ / R_{ij} (R_c)	W	N	P	G	C	K	L
**W**	25.0/ 1.0	19.0/ 1.0953	22.4/ 1.0371	26.1/ 0.9857	62.0/ 0.9912	22.0/ 1.0	62.0/ 1.0
**N**		22.9/ 1.1907	22.0/ 1.1325	23.0/ 1.0811	40.0/ 1.0866	25.0/ 1.0	40.0/ 1.0
**P**			23.2/ 1.0743	26.0/ 1.0229	40.0/ 1.0984	25.0/ 1.0	42.0/ 1.0
**G**				27.3/ 0.9715	42.0/ 0.9770	25.0/ 1.0	40.0/ 1.0
**C**					30.2/ 0.9824	60.0/ 1.0	25.0/ 1.0
**K**						30.0/ 1.0	30.0/ 1.0
**L**							30.0/ 1.0

Table S2. The relative mass, charge and bonded interaction parameters of DPPC and water beads

Bead	Relative mass	Charge(e)	Bond	$l_b^{ij}(R_c)$	Angle	Θ_{ikj} (degrees)	$k_a^{ijk}(k_BT/$ rad^2
**W**	1.0	0	**N-P**	0.6	**N-P-G**	120	4.5
**B**	0.01	0	**P-G**	0.6	**P-G-G**	100	4.5
**N**	1.3531	+1	**G-G**	0.43	**P-G-C**	180	5.0
**P**	2.0167	-1	**G-C**	0.6	**G-G-C**	100	4.5
**G**	1.0462	0	**C-C**	0.516	**G-C-C**	180	5.0
**C**	0.786	0			**C-C-C**	180	5.0
**K/L**	1.0	0	K/L-K/L	0.65			

Table S3. DPD parameters of the exponential conservative force between the gas bead B and the other beads

	a_{BJ} (k_BT)	b_{BJ}	R_{BJ} (R_c)
**W**	43.2	10.0	0.98
**N**	43.2	10.0	1.05
**P**	43.2	10.0	1.05
**G**	43.2	10.0	1.0
**C**	36.0	2.0	0.98
**K**	43.2	10.0	0.98
**L**	36.0	2.0	0.98

S3. The JNP-DPPC monolayer simulation set up

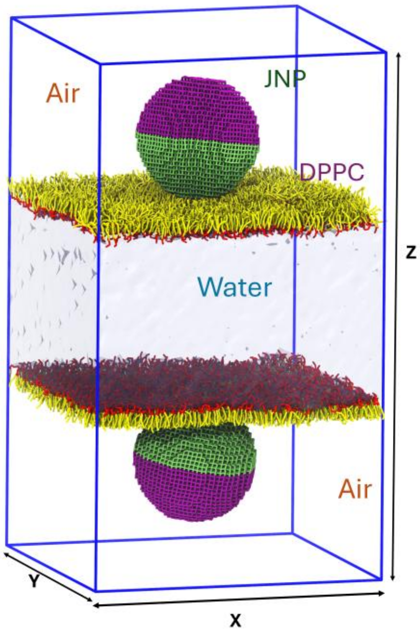

Figure S2. The simulation set up for the JNP-monolayer system. The gas beads B are not shown for clarity, and water is shown as blue transparent solvent phase. Other colors: purple-NP hydrophilic beads, lime-NP hydrophobic beads, red- lipid head groups, yellow-lipid tail groups.

Simulation systems are constructed with pre-equilibrated DPPC monolayer at $a_L = 0.6\ nm^2$ and JNPs as double monolayer systems with a water slab between the monolayers as shown in Figure S2. The system is periodic in X, Y and Z directions and thus, such an arrangement preserves symmetry across periodic boundaries. The details of the DPD simulations performed at different hydrophobic overages are provided in Table S4. The initial configuration of the systems is equilibrated with a short 20000 step NPT simulations imposing large mass (~ 50 times) on the NP and DPPC beads to make them immobile and to allow water and gas phases equilibrate. Following this, a 50000 step NPT

Table S4. Details of the simulated JNP-monolayer system.

JNP	*Lipophile adhesion (LA)*		*Hydrophile adhesion (HA)*	
Hydrophobic coverage (ϕ_L)	Total number of particles	System size (R_c^3)	Total number of particles	System size (R_c^3)
0			546274	$44.64 \times 44.64 \times 89.69$
0.1	546274	$44.64 \times 44.64 \times 89.68$	546274	$44.64 \times 44.64 \times 89.68$
0.25	546274	$44.65 \times 44.65 \times 89.7$	546274	$44.64 \times 44.64 \times 89.67$
0.40	546274	$44.63 \times 44.63 \times 89.66$	546274	$44.64 \times 44.64 \times 89.67$
0.5	486063	$44.65 \times 44.65 \times 79.73$	486063	$44.66 \times 44.66 \times 79.75$
0.6	546274	$44.63 \times 44.63 \times 89.66$	546274	$44.63 \times 44.63 \times 89.66$
0.75	546274	$44.63 \times 44.63 \times 89.65$	546274	$44.63 \times 44.63 \times 89.65$
0.9	546274	$44.63 \times 44.63 \times 89.64$	546274	$44.62 \times 44.62 \times 89.64$
1.0	546274	$44.63 \times 44.63 \times 89.65$		

simulation is performed with actual masses (see Table S2) to relax the system at pressure P=23.7. The temperature is set at $T_{DPD} = 0.65$, which corresponds to a real temperature $T_{real} =$ 293 K, following the temperature scaling approach developed in our previous work.[1]

Subsequently, simulations are run for 2-4 million steps at NVT conditions, until the average surface tension of the monolayer is constant and equilibrated. The JNPs are practically immobile during the first NPT equilibration with large masses. The NPs start to penetrate the monolayer during the second NPT and NVT simulations. Therefore, the time evolution of the systems is monitored during the second NPT equilibration and the subsequent NVT simulation.

S4. Surface Energy Calculations

S4.1 The radial distribution functions

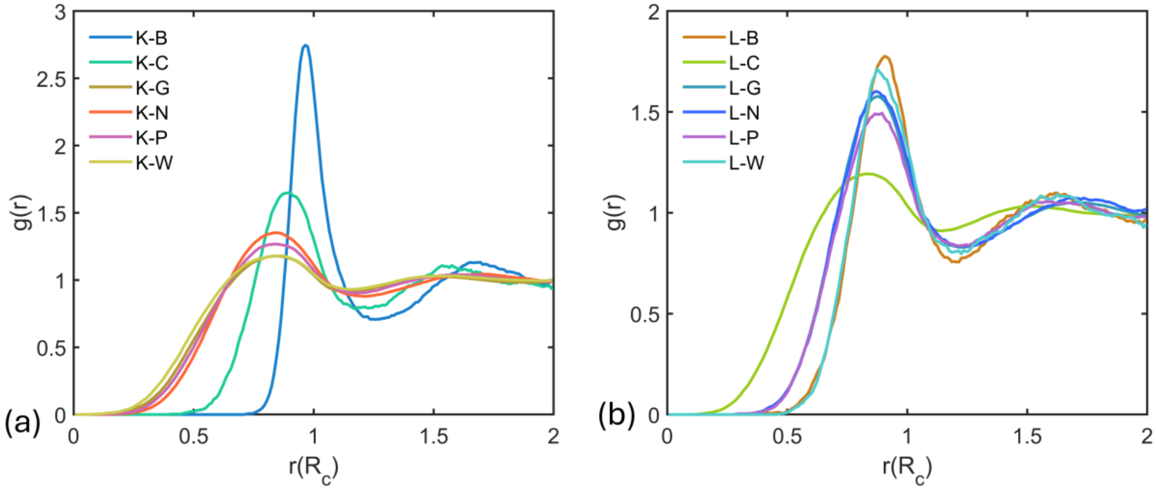

Figure S3. Radial distribution functions of the nanoparticle beads with other beads: (a) the hydrophilic bead K and (b) the hydrophobic bead L. The position of the valley after the first hydration shell (r_{valley}) lies beyond the interaction cutoff of 1 Rc.

Bead pair	ϵ_{Kj} (k_BT)	*Bead pair*	ϵ_{Lj} (k_BT)
K-B	0.5014	L-B	0.3872
K-C	0.4260	L-C	-0.1108
K-W	-0.1729	L-W	0.4294
K-N	-0.5109	L-N	-0.1846
K-G	-0.0465	L-G	0.2576
K-P	-0.2079	L-P	0.1405

Table S5. Bead-bead interaction energy of the nanoparticle beads with other beads.

To calculate the bead-bead contact energy, (Eq. (10) of the manuscript), the radial distribution functions $g_{\alpha j}(r)$ needs to be determined for each respective bead pair. For this, we simulated systems of free NP beads K and L in 'solvent' environments of the other bead types W, B, N, G, P and C. The systems contained 3000 beads with a few NP beads, K or L and the rest being one of the other bead types. NP beads are well solvated and remain far apart in a solvent of beads

with favorable interaction; for instance, K beads in water or L beads in a solvent consisting of C beads. However, in a repelling solvent environment, (such as K in C or L in W) NP beads tend to aggregate, which affects $g_{\alpha j}(r)$. In these cases, we chose a very small number (1-5) of NP beads to ensure that the NP beads are well surrounded by the solvent beads.

Figure S3 shows the radial distribution functions of the NP beads $g_{Kj}(r)$ and $g_{Lj}(r)$. The number of solvent beads surrounding the NP bead can be calculated by integrating $g_{\alpha j}(r)$ in the first hydration shell, marked by the valley minimum (r_{valley}) after the first peak. We find that in all cases, $r_{valley} > R_{\alpha j}$ and therefore Eq. (10) can be evaluated by integrating upto $R_{\alpha j}$, as V_{ij} is zero beyond it. The calculated $\epsilon_{\alpha j}$s are given in Table S5, which shows negative energies for favorable interactions and positive energies for unfavorable interactions.

S4.2 Potential energy of the exponential interaction

The exponential conservative force is given by,

$$F_{Bj}^{exp} = \frac{a_{Bj}}{1 - e^{b_{Bj}}} \left(e^{\frac{b_{BJ} r_{Bj}}{R_{Bj}}} - e^{b_{BJ}} \right). \tag{S1}$$

The corresponding potential is obtained by integration,

$$V_{Bj} = -\int F_{ij} dr_{Bj} = -\frac{a_{Bj}}{1 - e^{b_{Bj}}} \left(\frac{R_{Bj}}{b_{Bj}} e^{\frac{b_{BJ} r_{Bj}}{R_{Bj}}} - r_{Bj}\, e^{b_{BJ}} \right) + C\,, \tag{S2}$$

where C is the integration constant, which can be determined by setting,

$$V_{Bj}(R_{Bj}) = 0 = -\frac{a_{Bj}}{1 - e^{b_{Bj}}} \left(\frac{R_{Bj}}{b_{Bj}} e^{b_{Bj}} - R_{Bj}\, e^{b_{BJ}} \right) + C\,, \tag{S3}$$

which gives,

$$C = \frac{e^{b_{Bj}} R_{Bj} a_{Bj}}{1 - e^{b_{Bj}}} \left(\frac{1}{b_{Bj}} - 1 \right). \tag{S4}$$

Therefore,

$$V_{Bj}(r_{Bj}) = -\frac{a_{Bj}}{1 - e^{b_{Bj}}} \left(\frac{R_{Bj}}{b_{Bj}} e^{\frac{b_{BJ} r_{Bj}}{R_{Bj}}} - r_{Bj}\, e^{b_{BJ}} \right) + \frac{e^{b_{Bj}} R_{Bj} a_{Bj}}{1 - e^{b_{Bj}}} \left(\frac{1}{b_{Bj}} - 1 \right) \tag{S5}$$

$$= \frac{a_{Bj}}{1 - e^{b_{Bj}}} \left((r_{Bj} - R_{Bj}) e^{b_{BJ}} + \frac{R_{Bj}}{b_{Bj}} \left(e^{b_{BJ}} - e^{\frac{b_{BJ} r_{Bj}}{R_{Bj}}} \right) \right) \tag{S6}$$

S5. JNP-induced Morphological Behavior

S5.1 Monolayer disruption and healing

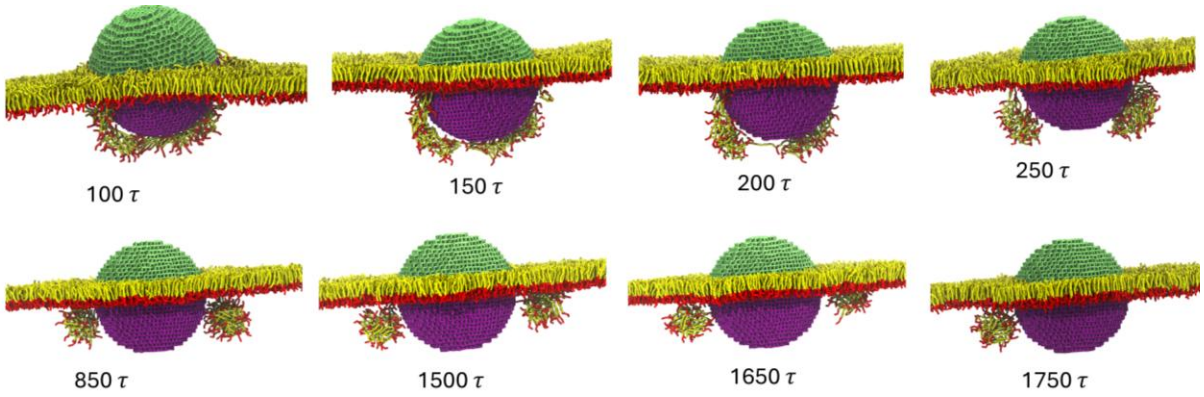

Figure S4. Disruption of the DPPC monolayer upon insertion of a JNP with 50% coverage in the HA mode. Lipids from the disrupted region form micelles, some of which re-adsorb onto the monolayer through micelle–monolayer fusion. One micelle remains in the bulk phase until the end of the simulation (4000 τ)

S5.2 Monolayer phase behavior

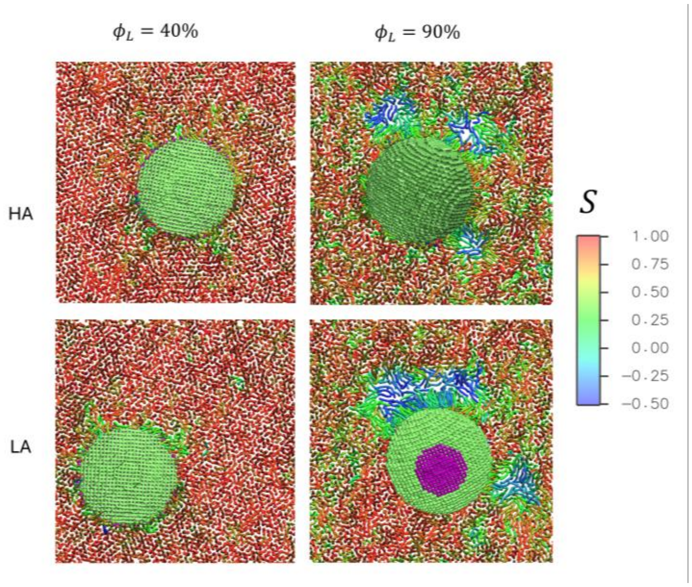

Figure S5. Top view of JNP adsorbed DPPC monolayers at hydrophobic coverages 40% and 90%. Lipids are colored according to their tail order parameter. JNP hydrophobic part colored lime and hydrophilic part purple.

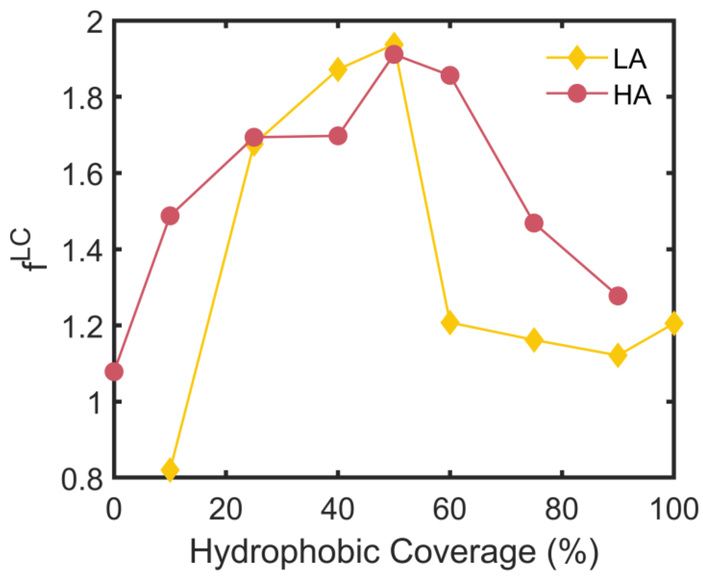

Figure S6. The number of lipids in the LC phase per effective area of the monolayers, as a function of hydrophobic coverage in the LA and HA modes.

References

1. Santo, K. P.; Iepure, M.; Arrendondo, J.; Min, Y.; Neimark, A. V., Temperature-dependent mechanical and phase behavior of pulmonary surfactant monolayers studied by dissipative particle dynamics modeling and experiments. *Colloids Surf. Physicochem. Eng. Aspects* **2025,** *725*, 137623. doi:https://doi.org/10.1016/j.colsurfa.2025.137623
